# Promoting Exercise Training Remotely

**DOI:** 10.3390/life12020262

**Published:** 2022-02-09

**Authors:** Stephanie A. Robinson, Marilyn L. Moy

**Affiliations:** 1 Center for Healthcare Organization and Implementation Research, VA Bedford Healthcare System, Bedford, MA 01730, USA; 2 The Pulmonary Center, Boston University School of Medicine, Boston, MA 02118, USA; 3 Pulmonary and Critical Care Medicine Section, VA Boston Healthcare System, Boston, MA 02130, USA; marilyn.moy@va.gov; 4 Department of Medicine, Harvard Medical School, Boston, MA 02115, USA

**Keywords:** chronic respiratory disease, technology, exercise, narrative review, access

## Abstract

There has been increased incentivization to develop remote exercise training programs for those living with chronic respiratory diseases, such as chronic obstructive pulmonary disease (COPD). Remote programs offer patients an opportunity to overcome barriers to accessing traditional in-person programs, such as pulmonary rehabilitation (PR). Methods to deliver exercise training remotely range in complexity and types of technological modalities, including phone calls, real-time video conferencing, web- and app-based platforms, video games, and virtual reality (VR). There are a number of studies demonstrating the effectiveness of these programs on exercise capacity, dyspnea, and health-related quality of life (HRQL). However, there is great variation in these programs, making it difficult to assess findings across studies. Other aspects that contribute to the effectiveness of these programs include stakeholder perceptions, such as motivation and willingness to engage, and adherence. Finally, while the intent of these remote programs is to overcome barriers to access, they may inadvertently exacerbate access disparities. Future program development efforts should focus on standardizing how remote exercise training is delivered, engaging stakeholders early on to develop patient-centered programs that patients will want to use, and understanding the heterogeneous preferences and needs of those living with chronic respiratory disease in order to facilitate engagement with these programs.

## 1. Introduction

Chronic respiratory diseases, such as chronic obstructive pulmonary disease (COPD), interstitial lung disease (ILD), bronchiectasis, chronic asthma, and pulmonary hypertension are leading causes of death and disability worldwide [1]. Collectively, individuals living with chronic respiratory disease experience breathlessness limiting functional capacity, impaired exercise tolerance, reduced health-related quality of life (HRQL), repeated hospitalizations, and increased prevalence of anxiety and depression. There is Level 1 evidence that pulmonary rehabilitation (PR) enables individuals with COPD to achieve clinically important gains in exercise and functional capacity, as well as symptom reduction and improvements in HRQL [2,3]. There is also growing evidence for the efficacy of PR to improve similar outcomes in other chronic respiratory diseases, including ILD [4], bronchiectasis [5], and pulmonary hypertension [6].

Exercise training is the foundation of PR and includes both aerobic and strength training. PR incorporates exercise training and disease education to improve the physiological and psychological condition of individuals with chronic respiratory disease [7]. Typically, PR is delivered in an outpatient, or community setting, with several sessions a week for eight weeks or more [8]. Session consists of 30 min of aerobic training, which often consists of walking and cycling to achieve a target heart rate [9,10]. Strength training is focused on both the upper and lower limbs and uses repetitive lifting of loads to produce fatigue after eight to 12 repetitions [11,12,13].

Despite the evidence base supporting center-based PR, the proportion of patients who are referred to PR, complete the program, and maintain exercise adherence after completion remains low. Barriers such as lack of transport, geographic distance to a program, limited mobility, and intercurrent medical illness are widely accepted limitations to PR uptake and attendance in people with COPD [14]. Additionally, as a result of the COVID-19 pandemic, many PR programs shut down, further exacerbating access barriers. To increase access to the benefits provided by traditional, center-based PR and promote long-term adherence to exercise, there has been increased incentivization to develop effective, remote interventions. These remote interventions (i.e., telehealth interventions) refer to interventions that are delivered at a distance through the use of telecommunications or virtual technology [15]. Delivering exercise training to patients remotely, via technology, has the potential to overcome significant barriers to participation in PR. Technology-based exercise training may range in its technical complexity and utilize a number of technological modalities, including phone calls, real-time video conferencing, web- and app-based platforms, video games, and other cutting-edge technology such as virtual reality.

### Objective

This narrative review aims to synthesize recent assessments of remote exercise training programs. To facilitate our review, we conducted a literature search in PubMed. We immediately excluded any publications which did not include exercise training in the intervention, those which were not trials of some nature (i.e., pilots and randomized controlled trials (RCTs) were both included, but protocol papers, reviews, and editorials were not), and those that were published before 2011. We conducted an exploratory literature search within the review articles we identified and searched for primary outcomes papers for the protocol papers we identified. We focused our synopses of each study on exercise capacity, dyspnea, and HRQL, when available. These studies are summarized in Table 1. This review also explores other factors, such as stakeholder perceptions, and future directions that can support the clinical uptake of such programs. We discuss the need to screen patients for safety and eligibility as well as the limitations of remote exercise training.

## 2. Types of Remote Training Program

### 2.1. Telephone

Remote exercise training via telephone is perhaps the simplest type of technology modality. In contrast to other technology-enabled interventions (detailed below), interventions that rely on telephones are likely able to reach more participants as phone ownership is ubiquitous compared to ownership of more advanced technologies such as a smartphone [30]. Holland and colleagues assessed whether home-based PR, including exercise training and self-management education in patients with COPD, resulted in equivalent outcomes to traditional, center-based PR [16]. Remote exercise training was performed using equipment and activities that were easily accessible in the home, including sit-to-stand from a chair, step ups on a step, and water bottles for upper limb weights. After an initial home visit by a physiotherapist, participants were followed up over 7 weeks with weekly phone calls, which used motivational interviewing to build motivation for exercise. HRQL related to dyspnea and fatigue, measured by the Chronic Respiratory Questionnaire (CRQ), significantly improved compared to conventional PR. The telephone-based program did demonstrate similar improvements in exercise capacity (6-min walk test distance [6MWD] of 18.6 m) compared to those who participated in conventional PR [16]. However, when comparing this phone-based intervention to usual care (7 weekly social calls), the authors did not find any significant differences between groups in exercise capacity [17]. However, in the trial comparing phone-based home PR to usual care, the participants had a markedly higher baseline level of exercise capacity compared to their earlier trial [17]. The authors speculated that the phone-based intervention may not have been sufficient to impact outcomes in these relatively healthier patients. Not all remotely delivered interventions are aligned with the heterogeneous needs of individuals living with COPD, and some patients may require a greater push (e.g., targeted physical activity counselling) to elicit clinical improvements. Therefore, despite the potential wide reach of telephone-only interventions, they may need extensive personalization to be sufficient at eliciting behavior change across the varying needs of patients with COPD.

### 2.2. Videoconferencing

The majority of remote exercise training programs have used real-time videoconferencing to enable participants to synchronously view and talk to health professionals and/or other participants via a video-enabled screen [31]. Videoconferencing, via computer, laptop, tablet, or mobile device, can be used to deliver exercise training directly to the patient in their own home, or at a location that is more convenient than traveling to a hospital-based program. In a superiority trial, Hansen and colleagues compared videoconferencing-delivered pulmonary tele-rehabilitation to conventional PR in COPD [18]. Using videoconferencing software, pulmonary tele-rehabilitation was delivered in a group-based and supervised program to patients in their homes three times per week for 10 weeks. Exercise sessions included a warm-up and high repetitive time-based muscle endurance training. These exercises used bodyweight and dumbbells and a step box and included lower and upper body exercises such as sit-to-stand and shoulder presses. At both 10 weeks [18] and a 12-month follow-up [32], there were no significant differences in exercise capacity between tele-rehabilitation and conventional PR. HRQL, measured with the EuroQol 5-Dimension Questionnaire, did not significantly or clinically improve in either group. Notably, the pulmonary tele-rehabilitation program had a higher rate of completion compared to conventional PR [18].

In another superiority randomized controlled trial, participants with COPD were randomized to receive either a supervised home-based tele-rehabilitation program with exercise training three times a week for 8 weeks, or a usual care group without exercise training [19]. Participants in the tele-rehabilitation group were given a laptop with video conferencing abilities and a stationary lower limb cycle ergometer, which were accompanied with a booklet and face-to-face education session on how to use the equipment. The tele-rehabilitation group showed significantly increased exercise capacity on the endurance shuttle walk test time compared to the usual care group (mean difference = 340 s) [19]. There were no significant within-group improvements or between-group differences in HRQL (measured via CRQ). Another non-inferiority randomized trial compared the efficacy of an online program (“myPR”) to conventional PR in COPD [20]. After a brief face-to-face introductory session, participants were instructed to access myPR which contained incremental exercise designed to be delivered in real time to the patient over videoconferencing. Exercises were identical in both myPR and conventional PR, including bodyweight resistance movements, such as biceps curls, squats, pushups along a wall, and seated leg extensions. This program also included educational videos on disease management. After six weeks, the authors found that the adjusted mean difference for exercise capacity (6MWD) was 23.8 m, which was above the non-inferiority threshold of –40.5 m [20]. Similarly, scores on the St. George’s Respiratory Questionnaire (SGRQ), a measure of disease-specific HRQL, suggested non-inferiority for the myPR group [20].

Another alternative to increase access to exercise training via videoconferencing is to deliver the intervention from the PR center to a satellite center that is more accessible to the patients. In this model, both the in-person and remote sites receive the same exercise training. Evaluations of this “hub-and-spoke” model have been promising. One pilot evaluated the effectiveness of such a model in COPD [21]. Under direct observation of the staff at the hub site through videoconferencing, the spoke (i.e., videoconferencing) sites used a physiotherapy technician and respiratory nurse to help monitor safety and prepare gym equipment at the spoke facilities. Exercise capacity and dyspnea significantly improved at both locations after seven weeks, compared to baseline. Additionally, exercise capacity, as measured via the incremental shuttle walk test, improved significantly more at the spoke site (137 m) compared to the hub site (124 m) [21]. The authors noted that the hub site did have a lower mean shuttle walk test at baseline which may suggest that clinical teams were more likely to refer healthier participants to the spoke site [21]. This pilot did not asses HRQL. In another study, Stickland and colleagues compared videoconferencing at a satellite center with supervision to traditional PR [22]. After 8 weeks, exercise capacity and health-related quality of life improved similarly in both groups. Exercise capacity, as assessed by the 12-min walk test, improved by 81 and 82 m in the telehealth and traditional PR groups, respectively. HRQL, as measured by the St. George’s Respiratory Questionnaire score, improved by 4.5% and 4.1% in the telehealth and traditional PR groups, respectively [22].

### 2.3. eHealth and mHealth

eHealth (i.e., electronic health) and mHealth (i.e., mobile health) interventions are platforms or interventions delivered via the internet (e.g., website) or mobile platforms (e.g., mobile apps). As internet access and smartphone ownership continue to increase [33], eHealth/mHealth solutions are becoming increasingly popular. Such technologies assist with automatic recording of patient-generated health data and exercise activities, such as heart rate and energy expenditure. These data can be provided directly to the patient. Additionally, certain platforms can send this patient-generated data to a central server, where health care professionals can monitor patients and provide feedback [34].

*e*Health refers to health services delivered through information and communication technology, such as computers or mobile phone, whereas *m*Health typically refers to platforms that incorporate the use of smart or portable devices. Many *e*Health exercise training interventions leverage web-based interventions to deliver exercise training remotely without direct supervision by providers. These web-based interventions, accessed anytime and anywhere by the patient, typically involve multiple components to encourage engagement [35]. Chaplin and colleagues compared the efficacy of a web-based PR program to conventional PR [23]. The web-based intervention included a home-based exercise program that incorporated both aerobic and strength training. Aerobic training included walking for a specified time at 85% of the participant’s baseline performance on the maximal shuttle walking exercise test. Strength training consisted of upper and lower limb resistance training with hand-held weights. After 6–7 weeks, both the web-based intervention and conventional PR groups significantly improved on the endurance shuttle walk test from baseline (189 s compared to 184.5 s), although there were no between-group differences [23]. This pattern was similar when HRQL (measured with the CRQ for dyspnea) was examined; both groups significantly improved, but there were no between-group differences in improvement. A randomized pilot trial investigated the use of and satisfaction of a web-based program that included four components: (1) activity coach for physical activity monitoring and real-time coaching of daily activity behavior, (2) web-based exercise program for home exercising, (3) self-management of COPD exacerbations via a triage diary on the web portal, including self-treatment of exacerbations, and (4) teleconsultation [24]. The exercise program consisted of breathing and relaxation exercises, as well as mobilization, resistance, and endurance training. Each participant received an individualized exercise prescription, created by the patient’s physiotherapist. The authors did not evaluate change in exercise capacity nor HRQL. Adherence to the exercise program was low; only 21% of the prescribed exercises were completed [24]. The authors hypothesized that perhaps both the exercises prescribed and the technology used were not sufficiently motivating or stimulating for at-home exercise and suggested that future work should incorporate motivational strategies (e.g., gaming technologies) and exercise variation to improve motivation [24]. A recent RCT evaluated whether use of an app after conventional PR was effective in maintaining the benefits achieved during PR [25]. Patients were provided with dumbbells and a stationary bike, and an app that guided them through aerobic and resistance exercises. While there were improvements in exercise capacity (mean 6MWD change = 19.9 m) and HRQL (measured with the CRQ), these improvements were not significant [25].

Use of eHealth and mHealth for exercise training has been extended to lung transplant candidates and recipients. Wickerson and colleagues performed a program evaluation of a web-based, remote-monitoring app [26]. This multicomponent app includes patient education, prompts and reminders, satisfaction and symptom surveys, biometric data monitoring which push alerts to a clinician dashboard, personalized care plans including a daily individualized exercise pathway, and asynchronous in-app texting and videoconferencing between patients and the health care team. Aerobic and resistance exercises were individually tailored to the patient’s oxygen requirements, disease stability, exercise capacity, and access to home exercise equipment. Participants were asked to perform the exercises at home, unsupervised, at least three times per week. Compared to historical data of the traditional center-based program, functional outcomes were lower in the tele-rehabilitation program. Specifically, exercise capacity (6MWD) decreased by 39 m. However, the authors speculated that ineffective implementation may have impacted the effectiveness of the tele-rehabilitation program [26].

### 2.4. Video Games and Other Technologies

There has been an exponential increase in studying video games [36]. These novel technologies offer a potentially more enjoyable and/or motivating means to deliver exercise training (i.e., exergaming). Video games can use motion detection technology to project the player’s movements on a screen. The intensity of exercise provided by the video games can vary widely and can include strength training (e.g., bodyweight or weighted movements), aerobics (e.g., jogging or boxing), balance games (e.g., yoga or skiing), or virtual sports (e.g., bowling or baseball) [37]. Studies that have investigated the use of videogames to deliver exercise training have either developed their own game or evaluated the effectiveness of commercial or recreational games that have been adapted by clinicians and researchers for use in rehabilitation [38]. As an example, a randomized controlled trial in children with moderate to severe asthma evaluated the impact of a video game compared to a traditional PR tool (treadmill) [27]. The authors used a commercially available track-and-field inspired game called “Reflex Ridge”. After 8 weeks of two 30-min sessions, both the gaming and treadmill groups demonstrated improvement in maximum aerobic capacity (measured by rate of oxygen uptake [VO_2_]). The gaming group did demonstrate significantly greater improvements in predicted maximum heart rate compared to the treadmill group. Another study evaluated the effectiveness of Wii Fit^TM^ Nintendo as a PR tool [28]. In a randomized controlled trial, patients with COPD were randomly assigned to use either the Wii Fit, which included yoga, strength training, and aerobic activity, or a control group (cycle ergometer) twice a week for 6 weeks. 6MWD improved from baseline significantly within both the control group (66.8 m) and the intervention group (52.4 m), although there were no between-group differences. Similarly, HRQL (via SGRQ) significantly improved in both groups but did not differ between groups. Dyspnea (via the Medical Research Council (MRC)) did not significantly improve within either group [28].

Incorporating virtual reality (VR) into video games is another emerging field. An increasing number of studies show encouraging results demonstrating feasibility, acceptability and safety of VR for exercise training for COPD [39]. A recent randomized controlled trial in COPD evaluated whether pairing virtual reality with exercise training was superior to exercise training alone [29]. In this study, exercise training included traditional PR and a stationary cycle ergometer. VR sessions included minigames focused on lower and upper body balance, strength, and endurance. Results found that VR paired with exercise training improved 6MWD from baseline by 39 m compared to 16 m in those in which you participated in exercise training alone. While it is hypothesized that gaming interventions may motivate participants more than traditional technology-enabled interventions, there remains limited evidence for adherence and effectiveness [38].

### 2.5. Involving Stakeholders

We have detailed many trials evaluating the effectiveness of remote technologies to deliver exercise training as part of research protocols. However, feasibility and acceptability studies also offer valuable information as to how remote exercise training programs will be received by patients and their clinical teams. Acceptance of technologies by those who will use them is critical to the success of novel remote initiatives [40]. User perceptions can be evaluated using simple questionnaires or more in-depth qualitative methods. For example, Infarinato and colleagues developed a scale based on the Technology Acceptance Model (TAM [41,42]) and were able to demonstrate that patients’ intention to use their remote rehabilitation program depended on their perceived usefulness and ease of use of the program [43]. Houchen-Wolloff and colleagues leveraged qualitative methods to understand patient perceptions of their web-based self-management program [44]. Through interviews, they discovered that those who felt more comfortable with a computer were more willing to engage with the intervention and felt a stronger sense of motivation. Conversely, those who expressed more difficulty using computers were less likely to engage with the intervention, which reduced their motivation to participate in the program [44].

Evaluating user perceptions after an intervention has been developed provides valuable information regarding its potential impact. However, researchers should also consider involving stakeholders in the development stage of the intervention. This participatory research offers a collaborative approach between researchers and stakeholders (e.g., patients, clinical team members, organizational leadership) during the development of an intervention/program [45]. One recent study used a participatory research design to explore perspectives of community stakeholders during the development of an in-home pulmonary tele-rehabilitation program for African American and Latino COPD patients from underserved communities [46]. Through focus groups, they identified concerns of patients and other stakeholders, including ensuring that the intervention and recruitment materials included representative persons from the African American and Latino communities, and that having a combination of in-person interaction in addition to technology-based interaction is more meaningful than and preferable to exclusively technology-based interactions.

## 3. Considerations

While remote programs for exercise training appear to be largely beneficial to improving clinical outcomes in chronic respiratory disease, there remain limitations to this approach. Primarily, remote programs may not be the safest option for all patients. By nature, the remote aspect of these programs offers enhanced accessibility to exercise training support for patients who have difficulty accessing in-person care. Consequently, participation in remote programs may be dangerous if an adverse health event were to happen and the patient is unable to access proper medical care. As such, proper evaluation of patients and their risks is essential to identifying eligibility. The Global Initiative for Chronic Obstructive Lung Disease (GOLD) recommends a multidimensional assessment of patients that includes current symptoms, risk of future exacerbations, and lung function. A recent a review [47] developed an algorithm for navigating multidimensional indices commonly used in clinical practice, including BODE (Body-mass index airway Obstruction, Dyspnea, Exercise) and its various iterations [48], ADO (Age, Dyspnea, airway Obstruction) [49], DOSE (Dyspnea, Obstruction, Smoking, previous severe Exacerbations) [50], CODEX (Comorbidity, Obstruction, Dyspnea, previous severe Exacerbations) [51], and COTE (Co-morbidity Test) [52]. This algorithm, or similar decision-making processes, would be a beneficial addition to remote exercise training programs to evaluate patient eligibility and safety.

These remote training programs have the potential to overcome barriers to accessing in-person programs. However, they may also inadvertently contribute to the “digital divide” by creating new access barriers and exacerbate existing healthcare disparities. Patients with COPD who lack access to the internet tend to be older, have a lower income, less education, and comorbid mobility-related diseases [53]. As such, efforts should be made to support patients who may be less likely to enroll in a remote exercise training program. For example, hub-and-spoke programs such as those detailed above offer opportunities for patients to access programs without having to travel far, while not having to worry about broadband access and their own self-efficacy for technology. Additionally, hybrid effectiveness-implementation trial designs could be utilized to simultaneously evaluate the effectiveness of the intervention or program [54], but also identify or test implementation strategies to facilitate patient adoption of these programs.

## 4. Conclusions

Acknowledging the emergence of new PR models, recent work sought to achieve consensus on the essential components of PR [8]. Unsurprisingly, exercise training was included in the final model [8]. However, there remains a need for further standardization of how exercise training is delivered in these emerging, remote models. There are numerous technology-mediated means to deliver remote exercise training for patients with chronic respiratory disease. The format of the exercise training components in the studies summarized above, and in Table 1, vary in the types of exercise provided, the amount of supervision, and the duration. Additionally, these modalities range in complexity, including telephone calls, videoconferencing, *e*Health and *m*Health-based programs, videogames, VR, or a combination of any of the above (e.g., Wickerson et al. [26]). This narrative review is not intended to be a systematic review, and it is possible that relevant studies were not captured in our search. However, it is clear from this review that there is a breadth of research and development in this topic. Currently, there is no evidence to support that one mode of delivery is superior to another for promoting exercise training and relevant clinical outcomes in chronic lung disease [31]. To support the uptake of remote exercise training in chronic respiratory disease, it is important to standardize and define the key characteristics required for an effective program. This standardization will increase our confidence in the effectiveness of these programs and support their future implementation. While beyond the scope of the current review, future systematic reviews and meta-analyses may begin to provide clarity regarding effective modalities and intensities. Additionally, acknowledging the heterogeneous nature of chronic lung disease, it is important to emphasize that remote exercise training programs are not suitable for all patients. Future work on such programs should devote substantial effort to ensuring that patients are properly screened for eligibility.

The most effective technology-based program is one that is efficacious, safe, and will be used by its targeted population. Remote options for exercise training offer patients more flexibility for when and where they can perform the exercises. Increased flexibility likely supports better short- and long-term adherence to the program, resulting in improved outcomes. However, if the patient does not adhere to the program, it is impossible to distinguish why the program failed to improve outcomes. As such, evaluating stakeholder perceptions of these programs will offer valuable insight into the future uptake of these programs.

Finally, remote programs have traditionally been a means to overcome barriers to accessing in-person care. The unintended consequences of these programs should continue to be evaluated so that they do not contribute to further barriers and healthcare inequities. Certain home-based programs may be a better fit for patients with good connectivity and digital self-efficacy, whereas other more supervised programs may be a better alternative for those who do not have the resources or feel as comfortable navigating the technology.

## Figures and Tables

**Table 1:** Summary of remote exercise training programs included in this narrative review.

Author	Study Population	Study Design	*n*	Intervention vs. Comparison Description	Exercise Training Type(s)	Duration of Program	Valence in Change in Outcomes Compared to Comparison Group		
							Exercise Capacity	Dyspnea	Health Related Quality of Life
**Telephone**									
Holland et al. 2017 [16]	COPD	RCT	166	Home-based PR vs. Center-based PR	Unsupervised aerobic and resistance training	1 home visit, 7 weekly calls	+	+	+
Lahham et al. 2020 [17]	COPD	RCT	58	Home-based vs. Usual care	Unsupervised aerobic and resistance training	1 home visit, 7 weekly calls	=	+	+
**Videoconferencing**									
Hansen et al. 2020 [18]	COPD	RCT	134	Home-based PR vs. Center-based PR	Supervised resistance training	10 weeks	=	+	=
Tsai et al., 2017 [19]	COPD	RCT	37	home-based PR vs. usual care	Supervised aerobic and resistance training	8 weeks	+	n/a	+
Bourne et al., 2017 [20]	COPD	RCT	90	Home-based PR vs. Center-based PR	Unsupervised bodyweight resistance movements	6 weeks	=	n/a	=
Knox et al., 2019 [21]	Variety	Pilot	45	Spoke site PR vs. Hub site PR	Supervised aerobic exercise	7 weeks	+	=	n/a
Stickland et al., 2011 [22]	COPD	Non-randomized trial	409	Spoke site PR vs. Hub site PR	Supervised aerobic and resistance training	8 weeks	=	n/a	=
**eHealth and mHealth**									
Chaplin et al., 2017 [23]	COPD	RCT Pilot	103	Web-based PR vs. Center-based PR	Unsupervised aerobic and strength training	6–7 weeks	=	=	=
Tabak et al., 2014 [24]	COPD	RCT Pilot	29	Web-based self-management program vs. Usual care	Unsupervised, individualized aerobic and exercise training	13–49 weeks	n/a	n/a	n/a
Galdiz et al., 2021 [25]	COPD	RCT	94	Web and app-based PR vs. Usual care after PR	Unsupervised aerobic and resistance training	8 weeks	=	n/a	=
Wickerson et al., 2021 [26]	Lung transplant candidates and recipients	Program evaluation	108	Web and app-based Program vs. Historical usual care	Unsupervised aerobic and resistance training	4 weeks	-	n/a	n/a
**Video Games and Other Technologies**									
Gomes et al., 2015 [27]	Children with moderate to severe asthma	RCT	36	Video game vs. treadmill	Supervised aerobic and bodyweight resistance training	8 weeks	=	n/a	n/a
Sutanto et al., 2019 [28]	COPD	RCT	20	Video game vs. cycle ergometer	Supervised aerobic and strength training vs. supervised cycling	6 weeks	=	=	=
Rutkowski et al., 2020 [29]	COPD	RCT	106	PR with exercise training vs. PR with exercise training and VR training vs. PR with VR training	Supervised aerobic and resistance training	2 weeks	+	n/a	n/a

Notes. +: intervention group improved more than the comparison group; =: intervention group changed similarly to comparison group; -: intervention group did not improve as much as the comparison group. n/a indicates that the information about this outcome was not provided. COPD = chronic obstructive pulmonary disease; RCT = randomized controlled trial; PR = pulmonary rehabilitation; VR = virtual reality.

## Data Availability

No new data were created or analyzed in this study. Data sharing is not applicable to this article.
